# Construction of engineered yeast producing ammonia from glutamine and soybean residues (okara)

**DOI:** 10.1186/s13568-020-01011-9

**Published:** 2020-04-15

**Authors:** Yukio Watanabe, Kouichi Kuroda, Yuki Tatemichi, Takeharu Nakahara, Wataru Aoki, Mitsuyoshi Ueda

**Affiliations:** 1 Division of Applied Life Sciences, Graduate School of Agriculture, Kyoto University, Sakyo‑ku, Kyoto, 606‑8502 Japan; 2 Research & Development Division, Kikkoman Corporation, Noda , Chiba, 278-8601 Japan; 3 Core Research for Evolutional Science and Technology (CREST), Japan Science and Technology Agency (JST), Chiyoda-ku, Tokyo, 102-0076 Japan

**Keywords:** Ammonia, Cell surface engineering, *Saccharomyces cerevisiae*, Glutaminase, Food waste

## Abstract

Ammonia is an essential substance for agriculture and the chemical industry. The intracellular production of ammonia in yeast (*Saccharomyces cerevisiae*) by metabolic engineering is difficult because yeast strongly assimilates ammonia, and the knockout of genes enabling this assimilation is lethal. Therefore, we attempted to produce ammonia outside the yeast cells by displaying a glutaminase (YbaS) from *Escherichia coli* on the yeast cell surface. YbaS-displaying yeast successfully produced 3.34 g/L ammonia from 32.6 g/L glutamine (83.2% conversion rate), providing it at a higher yield than in previous studies. Next, using YbaS-displaying yeast, we also succeeded in producing ammonia from glutamine in soybean residues (okara) produced as food waste from tofu production. Therefore, ammonia production outside cells by displaying ammonia-lyase on the cell surface is a promising strategy for producing ammonia from food waste as a novel energy resource, thereby preventing food loss.

## Introduction

Seventeen sustainable development goals (SDGs) have been proposed by the United Nations to solve global problems. Two of these goals are aimed at producing affordable and clean energy to combat global warming (United Nations 2019). To mitigate climate change, the development of technologies for sustainably producing energy and chemicals from biomass has become increasingly important (Lopes 2015; Wernick and Liao 2013).

Ammonia is one of the most important chemicals for producing fertilizer and is essential for our daily lives. The production of various materials in the chemical industry, including plastics and pharmaceuticals, also requires ammonia (Erisman et al. 2008). Recently, attempts to establish a society based on hydrogen (hydrogen economy) have been developing rapidly. Ammonia is expected to act as a carrier of hydrogen (Lan et al. 2012; Miura and Tezuka 2014) because it has high hydrogen content (17.6%), no carbon, and high mass density. Moreover, ammonia has high storability and safety because there is little possibility of it exploding and it is easy to handle (Elishav et al. 2017; Valera-Medina et al. 2018). Therefore, the utility and demand for ammonia are increasing.

The Harbor–Bosch process has been used for over 100 years to produce enormous amounts of ammonia, more than 100 million tons per year. However, this process requires a large amount of fossil fuels, at present constituting more than 1% of the energy generated globally, and high pressures (150–350 atm) and temperatures (350 °C–550 °C) (Schrock 2006); this is because cleavage of the triple bond of nitrogen molecules requires a lot of energy. Fossil fuels are released into the atmosphere as greenhouse gases after being burned and cause environmental problems. In addition, most of the crops produced with fertilizers from ammonia are disposed as food waste. Landfilled food waste is converted into the greenhouse gases, nitrogen oxides (Galloway et al. 2008; Montzka et al. 2011). For example, over 3.9 million tons of soybean residues (okara) are produced every year as a by-product of the manufacture of soymilk and the Japanese traditional food tofu; most is landfilled or incinerated due to its low storability (Liu et al. 2016; Vong et al. 2016). Therefore, ammonia production from soybean residues would have a low environmental burden.

A biological method can be used for obtaining ammonia from biomass under milder conditions than in the Harbor–Bosch process. In recent years, many researchers have attempted to produce biofuels and chemicals from carbon-containing biomass by metabolic engineering (Kircher 2015; Liao et al. 2016; Rabinovitch-Deere et al. 2013; Shi and Zhao 2017). However, there are few reports of successful biological technologies for the sustainable utilization of nitrogen (Vong et al. 2016). Some bacteria producing ammonia from biomass were previously created with metabolic engineering (Choi et al. 2014; Mikami et al. 2017). Disruption of the genes related to assimilation and overexpression of the genes catabolizing amino acids were effective for producing ammonia. However, nitrogen compounds such as amino acids and ammonia are essential nutrients for microorganisms and can be used for their growth before their secretion outside of cells (Choi et al. 2014; Mikami et al. 2017). Moreover, a high concentration of ammonia is toxic to living organisms (Bittsanszky et al. 2015; Muller et al. 2006; Santos et al. 2012).

The yeast *Saccharomyces cerevisiae* is one of the most suitable organisms for producing substances because yeast remains stable under various environmental conditions such as low pH and has a low risk of contamination (Kuroda and Ueda 2016). Furthermore, yeast is already widely utilized in industry to produce high-value chemicals via many advanced manipulation techniques (Liu et al. 2019). However, there is a problem that the knockout of nitrogen assimilation-related genes to avoid assimilation causes lower growth of yeast as well as *E. coli* (Magasanik 2003). Therefore, we attempted to produce ammonia outside the cells at high yield by displaying an amino-acid-catabolizing enzyme using a cell surface engineering system (Kuroda and Ueda 2011; Kuroda and Ueda 2013; Ueda 2016) to avoid the ammonia assimilation and toxicity inside the cells. To display target proteins using cell surface engineering, they are fused to a secretion signal at the N-terminus and an α-agglutinin cell wall-anchoring protein including glycosylphosphatidylinositol (GPI) anchor attachment signal sequence at the C-terminus. Cell surface engineering enables yeast to display about 10^5^ to 10^6^ target proteins per cell on its surface and to be used directly as a whole-cell biocatalyst. In addition, the displayed enzymes are immobilized on the cell wall, which contributes to stabilizing them and making them reusable (Bae et al. 2015; Inokuma et al. 2014; Motone et al. 2016; Nakanishi et al. 2012; Ota et al. 2013; Takagi et al. 2016).

Amino-acid-catabolizing enzymes that produce ammonia from amino acids include amino acid deaminase and amino acid transaminase (Brown et al. 2008; Hartman 1968; Lu et al. 2013; Molla et al. 2017; Pollegioni et al. 2013); they require cofactors such as NAD and FAD for their reactions. However, these cofactors are generally expensive and need to be regenerated by other reactions. In contrast, ammonia-lyases do not require any cofactors for their activity. In particular, glutamine ammonia-lyase is suitable for displaying on the yeast cell surface because food contains numerous glutamine residues as a substrate for ammonia production (Eppendorfer and Bille 1996; Ninomiya 1998). Among the glutamine ammonia-lyases, we selected glutaminase from *E*. *coli* (YbaS), which is used by *E. coli* for neutralizing the gastric acid of the host to protect itself; its conversion efficiency from glutamine into ammonia is known to be high (Lu et al. 2013).

In this study, by displaying YbaS on the yeast cell surface, we attempted to produce ammonia from glutamine included in glutamine-containing solution and the food waste soybean residues. YbaS-displaying yeast produced a high yield of ammonia (3.34 g/L) from glutamine with a high conversion efficiency (83.2%). In addition, we showed that YbaS-displaying yeast also produced ammonia from the food waste soybean residues by avoiding assimilation. This study shows that ammonia production outside cells via cell surface engineering is effective for producing ammonia from food waste.

## Materials and methods

### Strains and media

*E. coli* strain DH5α [*F*^−^, ϕ80d*lacZ*ΔM15, Δ (*lac*ZYA-*arg*F) U169, *end*A1, *hsd*R17 (r_k_^−^, m_k_^+^), *sup*E44, *thi*-1, λ^−^, *rec*A1, *gyr*A96, *rel*A1, *deo*R] was used as the host for recombinant DNA manipulation and grown in Luria–Bertani medium [1% (w/v) tryptone (Becton, Dickinson and Company, MI, USA), 0.5% (w/v) yeast extract (BD), and 1% (w/v) sodium chloride] containing 100 μg/mL ampicillin (Meiji Seika Pharma, Tokyo, Japan).

*Saccharomyces cerevisiae* strain BY4741/*sed1*Δ (*MAT*a, *his3*Δ1, *leu2*Δ0, *met15*Δ0, *ura3*Δ0, *YDR077w::KanMX4*), obtained from EUROSCARF (Frankfurt, Germany), was used for the cell surface display of glutaminase (YbaS) from *E. coli* because *sed1*Δ resulted in an increase of the quantity of protein on the yeast cell surface (Kuroda et al. 2009). Yeast host cells were grown and transformed in synthetic complete without uracil (SC-Ura) medium [0.15% (w/v) yeast nitrogen base without amino acids and ammonium sulfate (BD), 0.5% (w/v) ammonium sulfate (Wako Pure Chemical Industries, Osaka, Japan), 0.19% (w/v) yeast synthetic drop-out medium supplements (Sigma-Aldrich, MO, USA), 2% (w/v) glucose]. For the production of ammonia by YbaS-displaying yeast, the transformants were cultured in SC-Ura buffered at pH 5.5 with 200 mM 2-morpholinoethanesulfonic acid (MES) (Nacalai Tesque, Kyoto, Japan).

### Pretreatment of soybean residues (okara)

Soybean residues (Nihon Beans, Tokyo, Japan), called okara, were obtained commercially. These residues [14% (w/w)] were diluted in 50 mM MES (pH 5.5) and sterilized. The enzymes described below were purchased from Amano Enzyme (Nagoya, Japan). The protease mixture contained 2 mg/mL ProteAX, Peptidase R, PROTIN SD-AV10, Protease M “Amano” SD, PROTIN SD-NY10, THERMOASE R PC10F, and Protease A “Amano” SD in 50 mM MES (pH 5.5). The cellulase mixture contained 2 mg/mL Hemicellulase “Amano” 90, Cellulase A “Amano” 3, Mannanase BGM “Amano” 10, Cellulase T “Amano” 4, and Pectinase G “Amano” in 50 mM MES (pH 5.5). The protease mixture and cellulase mixture were filtrated through a 0.45 μm PVDF filter (Merck Millipore, MA, USA) and poured into sterilized soybean residues. The mixture of soybean residues containing these enzymes was incubated at 55 °C for 72 h with shaking at 250 rpm (Bio-Shaker BR-300LF, Japan). For denaturing the enzymes, the mixture was incubated at 80 °C for 30 min. Then, the mixture was filtrated with ADVANTEC131 (3 μm particle retention capacity; Toyo Roshi Kaisha, Tokyo, Japan) after the filtration with ADVANTEC2 (5 μm particle retention capacity; Toyo Roshi Kaisha). After that, the flow-through was filtrated through a 0.45 μm PVDF filter (Merck Millipore) and used for the substrate of ammonia production.

### Construction of plasmid for cell surface display of glutaminase

The genomic DNA of *E. coli* K-12 (Baba et al. 2006) strain was extracted using GenElute Bacterial Genomic DNA Kit (Sigma-Aldrich) after growing in Luria–Bertani medium at 37 °C for 12 h. To amplify the glutaminase gene (*YbaS*) from *E. coli* genomic DNA by polymerase chain reaction (PCR), primers YbaS-F (5′-GTTTCTGCCAGATCTATGTTAGATGCAAAC-3′), YbaS-R (5′-AGATCCACCCTCGAGTCAGCCCTTAAACAC-3′), and KOD FX Neo (Toyobo, Osaka, Japan) were used. The DNA fragment of PCR-amplified *YbaS* was digested with *Bgl*II and *Xho*I, followed by insertion into the vector pULD1 (Kuroda et al. 2009). pULD1-s (Kuroda et al. 2009) is a pULD1 analog containing a strep-tag instead of a FLAG-tag, and it was used as a negative control of immunofluorescence labeling and ammonia production. The constructed plasmid was named pULD1-YbaS.

### Yeast transformation

The constructed plasmid was introduced into *S. cerevisiae* BY4741/*sed1*Δ by using the lithium acetate method (Ito et al. 1983) with the Frozen-EZ Yeast Transformation Kit (Zymo Research, CA, USA). The transformant was selected on SC-Ura plate medium at 30 °C for 2 days.

### Immunofluorescence labeling of yeast and fluorescence microscopy

To confirm the display of YbaS on the yeast cell surface, immunofluorescent labeling of the FLAG tag was carried out as follows. Detection of the fluorescence of Alexa Fluor 488-conjugated goat anti-mouse IgG antibody (Thermo Fisher Scientific, MA, USA) on the cell surface indicates that the enzyme-FLAG tag fusion is displayed on the cell surface because the FLAG tag is located between the enzyme and the cell-wall-anchoring domain. Cells were incubated in phosphate-buffered saline (PBS; pH 7.4) containing 1% bovine serum albumin for 30 min before immunostaining for blocking. A mouse monoclonal anti-FLAG M2 antibody (Sigma-Aldrich) was used at a dilution rate of 1:300. A mixture of cells and antibody was incubated for 1.5 h at room temperature with gentle shaking. After washing with PBS (pH 7.4), as the secondary antibody, Alexa Fluor 488-conjugated goat anti-mouse IgG antibody (Thermo Fisher Scientific) was used at a dilution rate of 1:300 at room temperature with gentle shaking for 1.5 h. After washing with PBS (pH 7.4), the cells were observed with an inverted microscope IX71 (Olympus, Tokyo, Japan) through a U-MNIBA2 mirror unit with a BP470-490 excitation filter, a DM505 dichroic mirror, and a BA510-550 emission filter (Olympus). Live images were obtained using the Aqua Cosmos 2.0 software (Hamamatsu Photonics, Shizuoka, Japan) to control a digital charge-coupled device camera (Hamamatsu Photonics).

### Ammonia production by glutaminase-displaying yeast

After pre-cultivation in SC-Ura buffered at pH 5.5 for 36 h at 30 °C, the yeast cell culture was centrifuged at 3000×*g* for 5 min at 4 °C. The harvested cells were washed with sterilized distilled water. The cells were suspended in sterilized distilled water and cell suspension was inoculated into 200 mM MES buffer (pH 5.5) containing 37.3 g/L glutamine or solution containing 85.2 mg/L glutamine from pretreated soybean residues to an OD_600_ of 1. The reaction mixtures in 1.5 mL microtubes were shaken at 1000 rpm (Bio-Shaker M·B-024, Japan) at 37 °C and sampled at each time point. The ammonia concentration in the supernatant was measured using F-kit Ammonia (J.K. International, Tokyo, Japan). The measurement was based on the 340 nm absorbance of NADH, which is converted to NAD^+^ by the reaction with 2-oxoglutarate and ammonia. The ammonia yields presented in this manuscript were obtained by eliminating the background ammonia concentration as shown below. In the case of concentrations of ammonia produced from glutamine solution, the minimum ammonia concentrations produced by each yeast cell harboring pULD1-YbaS (0 h) or pULD1-s (0 h) were subtracted from those by yeast cells harboring pULD1-YbaS (0, 6, 12, 24, 36, 48 h) or pULD1-s (0, 6, 12, 24, 36, 48 h), respectively. In another case of concentrations of ammonia produced from pretreated soybean residues, the minimum ammonia concentrations produced by yeast cells harboring pULD1-YbaS (0 min) or pULD1-s (20 min) were subtracted from those by yeast cells harboring pULD1-YbaS (0, 5, 10, 20, 40, 60 min) or pULD1-s (0, 5, 10, 20, 40, 60 min), respectively. The conversion efficiency from glutamine to ammonia was calculated as follows: (ammonia concentration at each time point/glutamine concentration at 0 min).

### LC–MS/MS quantification of glutamine and other amino acids

To perform accurate quantification of amino acids using LC–MS/MS, we added l-glutamine-^13^C_5_,^15^N_2_, and labeled amino acid standards [50 μg/mL algal hydrolysate amino acid mixture (U-^13^C, 97%–99%; U-^15^N, 97%–99%; Cambridge Isotope Laboratories CNLM-452-0.5)] as an internal standard (IS) into pretreated soybean residues after reacting with the cell surface engineered yeasts.

The residual glutamine was quantified as shown below. LC experiments were conducted using HPLC (Nexera System; Shimadzu, Kyoto, Japan). Chromatographic separation was achieved using a Scherzo SS-C18 column (100 mm × 3 mm, 3 μm; Imtakt, Kyoto, Japan) with gradient elution with a mobile phase composed of eluents A (0.1% formate in water, v/v) and B (100 mM ammonium sulfate and 40% acetonitrile in water, v/v). The mobile phase was programmed as follows: isocratic elution of 5% B for the first 3 min, followed by linear gradient elution of 5% −100% B from 3 to 8 min. After the solvent composition of 100% B had been held from 8 to 11 min and then changed to 5% B for the next 2 min, it was returned to its starting condition and held for re-equilibration. The supernatant of pretreated soybean residues incubated with YbaS-displaying yeast or yeast harboring pULD1-s (1 μL) was injected onto the column and the flow rate was 0.3 mL/min for the first 3 min and 0.6 mL/min for the next 10 min.

To measure glutamine from pretreated soybean residues, the mobile phase was programmed as follows: isocratic elution of 5% B for the first 5 min, followed by linear gradient elutions of 5%− 10% B from 5 to 10 min and 10%–100% B from 10 to 20 min. After the solvent composition of 100% B had been held from 20 to 23 min and then changed to 5% B for the next 2 min, it was returned to its starting condition and held for re-equilibration. The supernatant of pretreated soybean residues incubated with YbaS-displaying yeast or yeast harboring pULD1-s (1 μL) was injected onto the column. The flow rate was 0.3 mL/min for the first 6 min and 0.6 mL/min for the next 23 min. The column temperature was set at 40 °C throughout the analysis. Glutamine was quantified using the multiple reaction monitoring (MRM) mode by triple quadrupole mass spectrometry (LCMS-8060; Shimadzu) equipped with an electrospray ionization source (ESI). The MS conditions were as follows: electrospray voltage, 4.0 kV; capillary temperature, 300 °C; and sheath gas, N_2_, 10 L/min. Each parameter of the MRM mode was as shown below: precursor ion (m/z), 147.1; product ion (m/z), 56.2; Q1 pre-bias, −2 V; and collision energy, −32 V. The MS conditions of amino acids obtained from soybean residues are shown in Additional file 1: Table S1. IS of amino acids including l-glutamine-^13^C_5_,^15^N_2_, and labeled amino acid standards [50 μg/mL algal hydrolysate amino acid mixture (U-^13^C, 97%–99%; U-^15^N, 97%–99%; Cambridge Isotope Laboratories CNLM-452-0.5)] was spiked in the samples for quantification of amino acids obtained from soybean residues.

## Results

### Construction of yeast displaying glutaminase on the cell surface

**Fig. 1:**
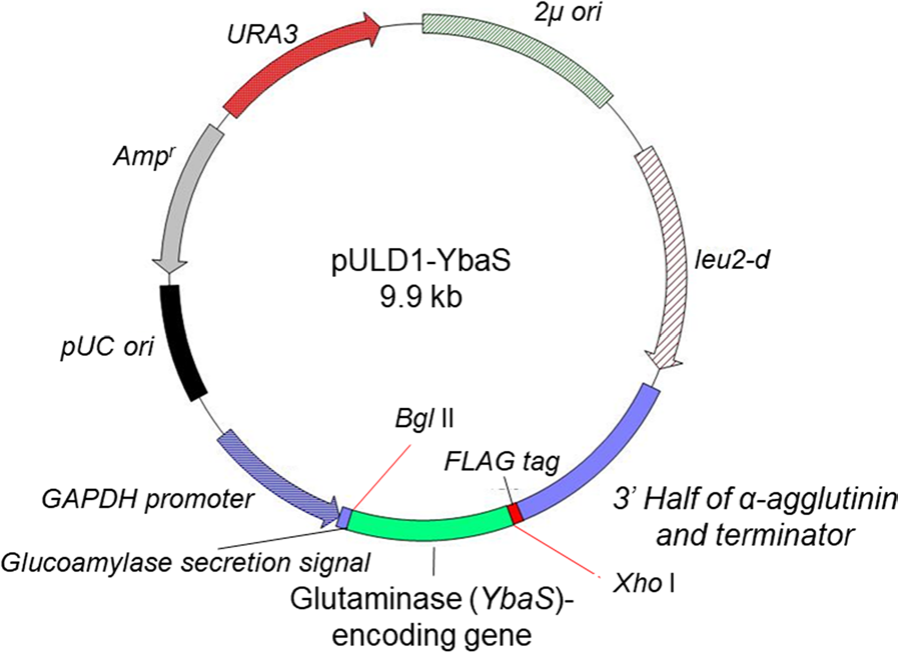
The constructed plasmid for the cell surface display of glutaminase from *E. coli* (YbaS)

**Fig. 2:**
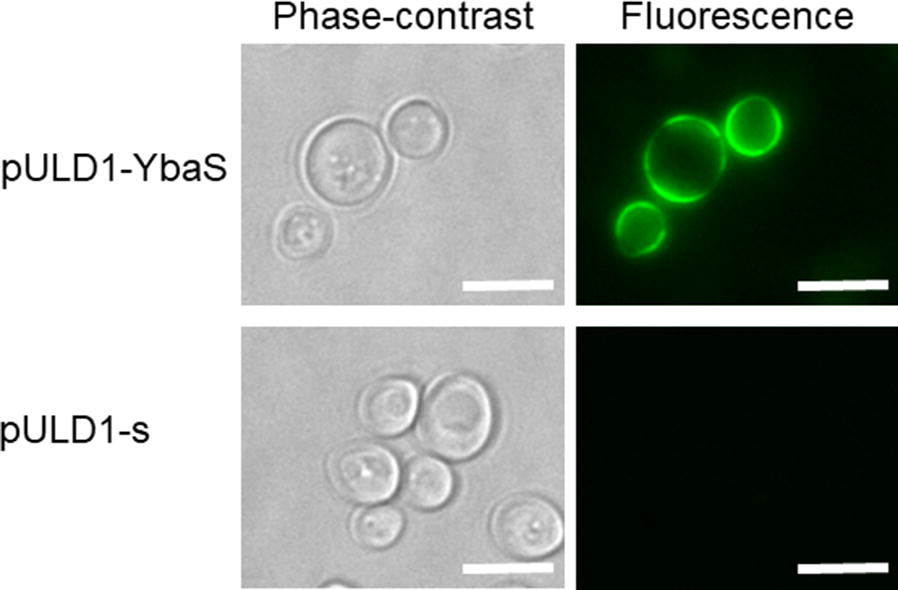
Fluorescence observation of cells after immunofluorescence labeling. An anti-FLAG antibody and Alexa Fluor 488 anti-mouse IgG antibody were used for labeling of the displayed proteins. Phase-contrast micrographs (left column), fluorescence micrograph (right column). The scale bars are 5 μm

To produce ammonia from glutamine outside the cells, we displayed glutaminase (YbaS), one of the amino-acid-catabolizing enzymes from *E. coli* (Lu et al. 2013), on the yeast cell surface. To display YbaS on the yeast cell surface, the glutaminase gene (*YbaS*) was inserted into pULD1, a cassette vector for the efficient display of proteins (Fig. 1). The constructed pULD1-YbaS was introduced into yeast. To confirm the display of YbaS on the yeast cell surface, immunofluorescence labeling was performed with mouse monoclonal anti-FLAG M2 antibody. The green fluorescence by Alexa Fluor 488 was observed on the yeast cell surface, indicating that the YbaS-FLAG tag fusion had been successfully displayed there. In contrast, no fluorescence was observed in yeast transformed with negative control plasmid, yeast harboring pULD1-s, for displaying only a strep-tag (Fig. 2).

### Ammonia production from glutamine by glutaminase displayed on the cell surface

**Fig. 3:**
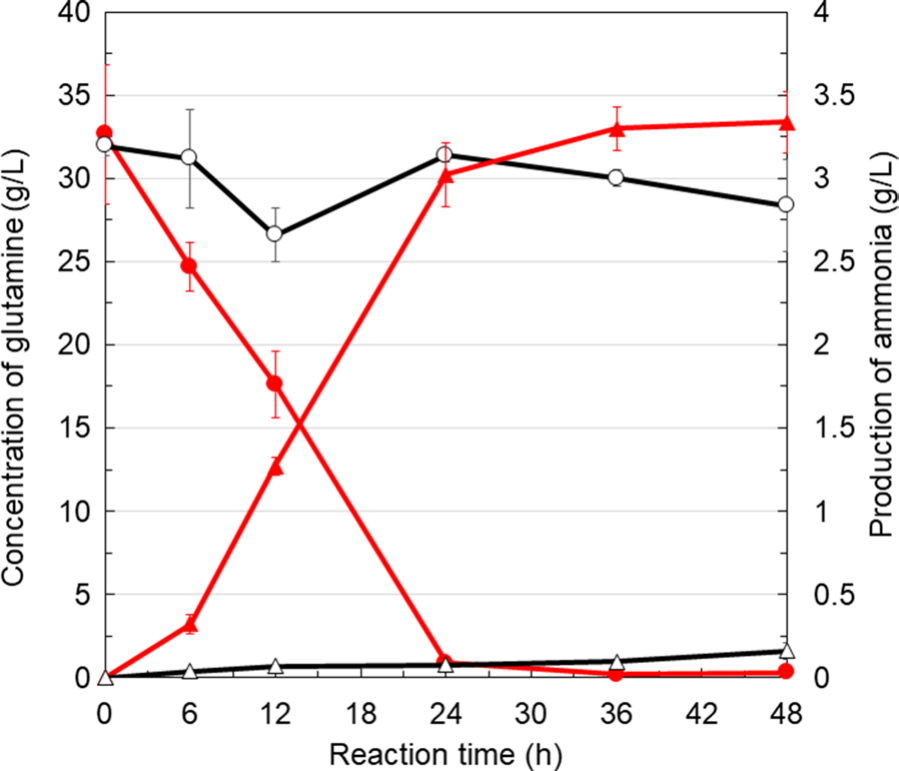
Ammonia production from glutamine by YbaS-displaying yeast. The amount of ammonia produced by YbaS-displaying yeast (pULD1-YbaS, red triangle) and control yeast (pULD1-s, open black triangle), and the residual glutamine by YbaS-displaying yeast (pULD1-YbaS, red circle) and control yeast (pULD1-s, open black circle) were measured. Values are given as mean ± SE (n = 3)

To detect the ammonia production by YbaS-displaying yeast on the cell surface, we measured the ammonia concentration in each sample by enzyme assay methods. YbaS-displaying yeast successfully produced 3.34 g/L ammonia at 48 h, whereas control yeast harboring pULD1-s produced almost no ammonia (Fig. 3). We monitored the glutamine converted by YbaS-displaying yeast with LC–MS/MS (Fig. 3). The obtained results suggest that YbaS-displaying yeast can produce ammonia from glutamine with high efficiency (83.2%).

### Ammonia production from pretreated soybean residues

**Fig. 4:**
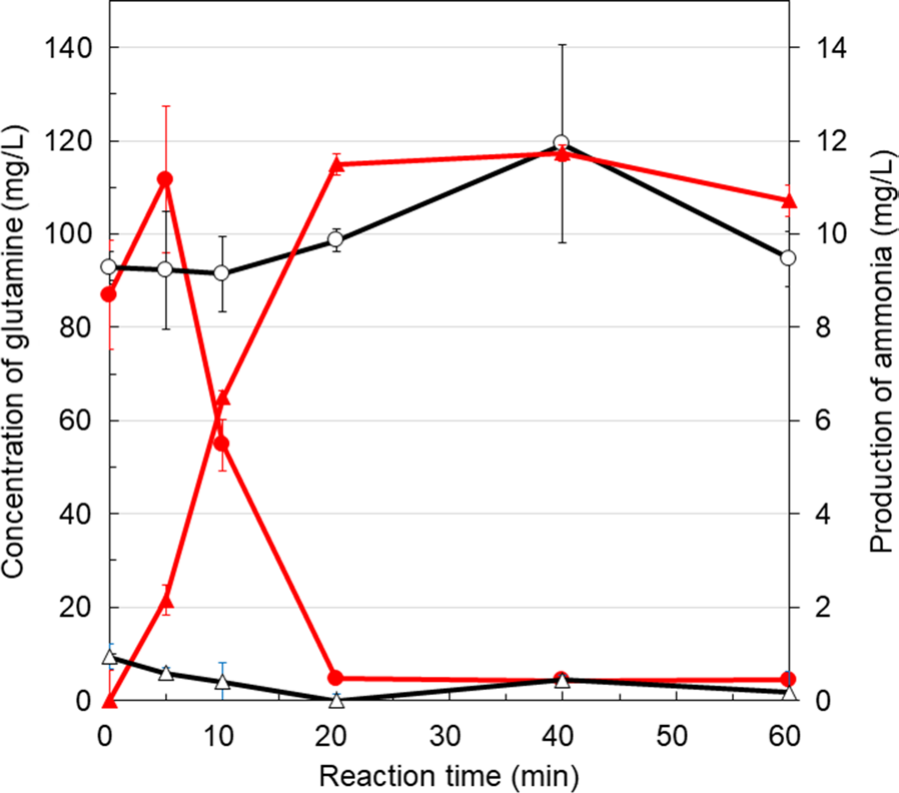
Ammonia production from pretreated soybean residues by YbaS-displaying yeast. The amount of ammonia produced by YbaS-displaying yeast (pULD1-YbaS, red triangle) and control yeast (pULD1-s, open black triangle), and the residual glutamine by YbaS-displaying yeast (pULD1-YbaS, red circle) and control yeast (pULD1-s, open black circle) were measured. Values are given as mean ± SE (n = 3)

Since we succeeded in producing ammonia from glutamine solution by YbaS-displaying yeast, we employed this yeast to produce ammonia from glutamine included in soybean residues pretreated with proteases and cellulases. These proteases degrade proteins contained in soybean residues and produce amino acids as products, and cellulases degrade cellulose associated with proteins and might increase the efficiency of protein degradation. YbaS-displaying yeast produced 11.7 mg/L ammonia from glutamine included in pretreated soybean residues at 40 min, whereas negative control, yeast harboring pULD1-s, produced almost no ammonia (Fig. 4). This indicates that YbaS-displaying yeast was able to produce ammonia from glutamine included in the pretreated soybean residues as food waste.

## Discussion

To establish a biological technique for efficient ammonia production, it is necessary to avoid ammonia assimilation that occurs inside yeast cells. In this study, we displayed glutaminase, YbaS, on the yeast cell surface to produce ammonia from glutamine included in pretreated soybean residues outside the cells. Figure 3 shows that YbaS-displaying yeast produced 3.34 g/L ammonia, which was 1.4 times and 7.3 times higher than the ammonia concentration in the previous studies by metabolically engineered *B. subtilis* and *E. coli* (Choi et al. 2014; Mikami et al. 2017). Therefore, we succeeded in producing high-concentration ammonia using yeast for the first time. In addition, the results indicate that the ammonia production by YbaS-displaying yeast was not affected by the toxicity of ammonia. A high concentration of ammonia above 0.1% (w/v) causes poor growth of yeast because of its toxicity, rather than an increase in pH (Santos et al. 2012). However, even though cells are damaged by the toxicity of ammonia, displayed enzymes could function and produce ammonia in our system because whether the cells are dead or alive does not affect the function of the enzyme displayed on the cell surface. Therefore, we have shown that extracellular ammonia production by cell surface engineering is effective to avoid ammonia assimilation and toxicity to yeast.

Previous studies on the production of ammonia by bacteria focused on intracellular production by metabolic engineering. For example, the yield of ammonia production by metabolically engineered *B. subtilis* was limited to 46.6% because ammonia is assimilated into the cell components (Choi et al. 2014). In contrast, we extracellularly produced ammonia from glutamine solution with high efficiency of 83.2% (Fig. 3). However, 83.2% conversion efficiency can be further increased in the future. In our experiment, the yeast in the reaction solution used may have been nitrogen-starved and may have taken up ammonia and glutamine. Since the transporter works mainly for uptake, the disruption of glutamine transporter (Schreve et al. 1998) or ammonia transporter (Marini et al. 1997) may contribute to further improvement of the production efficiency.

Furthermore, we showed that YbaS-displaying yeast can be applied to ammonia production from food waste. In this study, pretreated soybean residues were used as a representative type of food waste because soybean residues are produced during the process of making tofu. All of the glutamine from pretreated soybean residues were converted into ammonia by YbaS-displaying yeast and 122.0% conversion efficiency was achieved at 40 min (Fig. 4). The conversion efficiency exceeded 100% probably because ammonia in yeast cells leaked, or intracellular glutamine (30 mM, (Mulleder et al. 2016)) might leak and be converted to ammonia by YbaS on the cell surface. Furthermore, the glutamine concentration at 5 min was higher than that at 0 min in the samples reacted with YbaS-displaying yeast (Fig. 4). The reason for the increase in the glutamine concentration was not known, but this phenomena could contribute to the conversion efficiency exceeding 100%. These results indicate that the YbaS-displaying yeast can efficiently produce ammonia from food waste and that ammonia production by YbaS on the yeast cell surface was not inhibited by any other materials such as metal ions and sugars in pretreated soybean residues.

For producing ammonia from soybean residues, protein and cellulose in soybean residues were degraded by proteases and cellulases, and we quantified the concentrations of amino acids (Additional file 2: Figure S1). In a previous study, soybean residues were treated with a strong acid, but the pretreatment places a burden on the environment because of the need for post treatment (Kumar et al. 2016). In addition, glutamine was decomposed to glutamic acid by acid treatment in the previous study. In contrast, we used proteases and cellulases for degrading soybean residues under mild conditions (pH 5.5). We accurately quantified the concentrations of 16 proteinogenic amino acids, with the exceptions of cysteine, tryptophan, asparagine, and alanine (Additional file 2: Figure S1) from pretreated soybean residues. To compare the production of amino acids from proteins in the previous study, it was thought that the quantification of only essential amino acids other than tryptophan is sufficient (Kumar et al. 2016). Additional file 2: Figure S1 shows that the amount of glutamine produced from soybean residues was 84.8 mg/L. As a result, total essential amino acid content, excluding tryptophan produced from soybean residues in this study (Additional file 2: Figure S1), was 4.97 g/100 g. This was because proteases have specificity and it is difficult to completely convert all proteins to free amino acids, even if many different types of protease are used. To improve the conversion rate, it is efficient to optimize the amount and types of proteases and cellulases, or the reaction time and reaction temperature for degrading soybean residues. In the future, it is expected that all amino acids from soybean residues (Additional file 2: Figure S1) will be converted for ammonia production by the co-display of other ammonia lyases, contributing to the efficient utilization of food waste.

In this study, we showed the YbaS-displaying yeast produced high concentrations of ammonia from glutamine from pretreated soybean residues at high efficiency outside the cells. This is the first report describing an approach suitable to meet the SDGs of extracellular ammonia production from food waste by surface-engineered yeast that can avoid ammonia assimilation. Therefore, the yeast cell-surface-display system is useful for the efficient production of essential materials for cell growth or poisonous products at high concentration in yeast such as ammonia.

## Supplementary information

**Additional file 1: Table S1.** MRM conditions for the quantification of amino acids from soybean residues by LC–MS/MS. l-glutamine-^13^C_5_,^15^N_2_, and labeled amino acid standards [50 μg/mL algae hydrolysate amino acid mixture (U-^13^C, 97%–99%; U-^15^N, 97%–99%; Cambridge Isotope Laboratories CNLM-452-0.5)] as an IS were used for quantification of amino acids in pretreated soybean residues.

**Additional file 2: Figure S1.** Concentration of amino acids produced from soybean residues (n = 1). *Arg* arginine, *Asp* aspartate, *Gln* glutamine, *Glu* glutamate, *Gly* glycine, *His* histidine, *Ile* isoleucine, *Leu* leucine, *Lys* lysine, *Met* methionine, *Phe* phenylalanine, *Pro* proline, *Ser* serine, *Thr* threonine, *Tyr* tyrosine, *Val* valine.

## Data Availability

All relevant data are within the manuscript and its Supporting information files.

## Abbreviations

IS: Internal standard
CREST: Core research for evolutional science and technology
ESI: Electrospray ionization
JST: Japan science and technology
MRM: Multiple reaction monitoring
PCR: Polymerase chain reaction
