# Supplementary figures and images for: Construction of engineered yeast producing ammonia from glutamine and soybean residues (okara)
Source: AMB Express. 2020 Apr 15;10:70. doi: 10.1186/s13568-020-01011-9 (PMC7158961; doi:10.1186/s13568-020-01011-9)

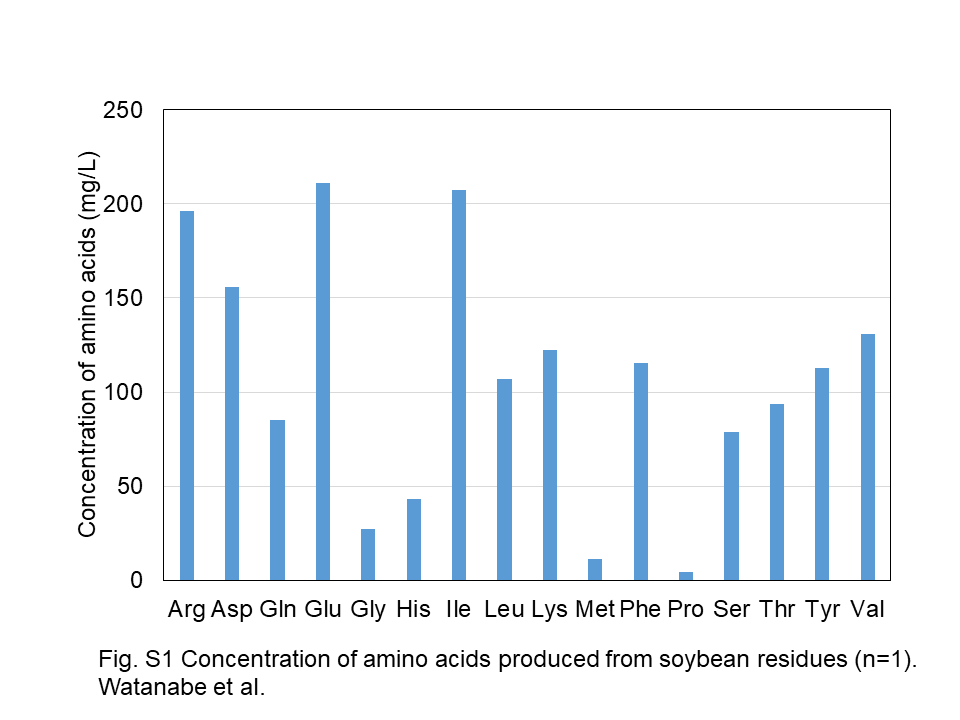

Supplement: Supplementary file 2 — Additional file 2: Figure S1. Concentration of amino acids produced from soybean residues (n = 1). Arg arginine, Asp aspartate, Gln glutamine, Glu glutamate, Gly glycine, His histidine, Ile isoleucine, Leu leucine, Lys lysine, Met methionine, Phe phenylalanine, Pro proline, Ser serine, Thr threonine, Tyr tyrosine, Val valine. [file 13568_2020_1011_MOESM2_ESM.tif]
